# Epidemiological characteristics and risk factors analysis of multidrug-resistant tuberculosis among tuberculosis population in Huzhou City, Eastern China

**DOI:** 10.1515/biol-2025-1081

**Published:** 2025-03-22

**Authors:** Haiyan Chen, Zhaowei Tong, Jianfeng Zhong, Qingqiu Zeng, Bin Shen, Fuchu Qian, Xin Xiao

**Affiliations:** Department of Infection, Huzhou Central Hospital, Huzhou Key Laboratory of Precision Medicine Research and Translation for Infectious Diseases, 1558 Sanhuan North Road, Wuxing District, Huzhou, Zhejiang, 313000, China

**Keywords:** pulmonary tuberculosis, multidrug-resistant tuberculosis, epidemiology, risk factors

## Abstract

The aim of this study was to examine the prevalence of multidrug-resistant tuberculosis (MDR-TB) within the tuberculosis (TB) population in Huzhou City, identify associated risk factors, furnish reference values for clinical practice, and establish standardized anti-TB treatment regimens. Retrospectively analyzing data from TB patients in Huzhou City encompassed 2,261 cases with sputum smear positive and 1,754 cases with sputum smear negative from January 2016 to December 2022. There were 3.66%(147/4,015) TB patients identified as MDR-TB. Multivariate logistic regression analysis showed that the risk of developing MDR-TB in individuals with comorbidities was 9.17 times higher than in individuals without comorbidities (odds ratio [OR] = 9.17, 95% confidence interval [CI]: 6.5–12.93, *P* < 0.001). The risk of progressing to MDR-TB with a positive sputum smear was 1.93 times higher than with a negative one (OR = 1.93, 95% CI: 1.35–2.77, *P* < 0.001). The risk of developing MDR-TB was 1.69 times higher in patients had smoking history than in those without (OR = 1.69, 95% CI: 1.11–2.56, *P* = 0.014). The main risk factors for developing MDR-TB were male patients, smoking history, sputum smear positive, farmer, body mass index ≤18.5, retreated cases, and those combination of diabetes or pneumoconiosis.

## 1 Introduction

Tuberculosis (TB) is one of the most significant infectious diseases that pose a severe threat to global public health. As reported in the 2022 World Health Organization Global Tuberculosis Report [1], in 2021, TB was responsible for approximately 1.6 million deaths worldwide. The incidence of rifampicin-resistant tuberculosis (RR-TB)/multidrug-resistant TB (MDR-TB) was estimated at around 450,000 new cases. TB has escalated to become the second-largest single-source cause of mortality, following coronavirus disease 2019 [2].

China ranks among the top seven nations globally in terms of the burden of MDR-TB, accounting for approximately two-thirds of MDR/RR-TB cases worldwide [1]. MDR-TB significantly contributes to the treatment failure of newly diagnosed TB cases and is associated with a high mortality rate. Early detection of drug resistance and effective treatment are paramount in controlling MDR-TB [3,4].

The clinical and molecular characteristics of drug-resistant strains in China exhibit regional disparities, as previously reported [5,6]. The national average drug resistance rate among new TB patients stands at 7.10%, whereas it escalates to 23.0% among retreated cases [7]. The current dearth of information regarding the prevalence of MDR-TB and extensively drug-resistant TB (XDR-TB) in the local region necessitates an understanding of the extent of drug-resistant TB (DR-TB) in the area. This understanding can facilitate the identification of pertinent risk factors, serve as a benchmark for clinical practice, and pave the way for the establishment of standardized anti-TB treatment regimens.

## 2 Materials and methods

### 2.1 Data collection

During the period from January 2016 to December 2022, we conducted a retrospective analysis of 4,015 cases of TB (including 2,261 cases of sputum-positive or molecular biology-positive pulmonary TB and 1,754 cases of sputum-negative pulmonary TB) in Huzhou City, Zhejiang Province. This study involved the collection of relevant clinical data for these cases from the hospital’s infectious disease reporting network and electronic medical record system. The collected information included (1) demographic characteristics: gender, age, occupation, residence, hygienic condition, poverty, etc., and (2) other factors: history of TB treatment, comorbidities, history of smoking, year of testing, sputum smear or molecular biology results, *Mycobacterium tuberculosis* culture results, molecular drug sensitivity results, drug-resistant mutation sites, etc.


**Informed consent:** Informed consent has been obtained from all individuals included in this study.
**Ethical approval:** The research related to human use has been complied with all the relevant national regulations and institutional policies and in accordance with the tenets of the Helsinki Declaration and has been approved by the authors’ institutional review board or equivalent committee.

### 2.2 Classification and definitions

Patients who underwent TB T-SPOT testing, sputum testing, and radiological examinations were classified into the following groups: (1) those with sputum-negative TB but a history of close contact with infectious TB cases displaying corresponding clinical symptoms (e.g., cough, sputum production, low-grade fever, and night sweats), or those with abnormal shadows or active TB changes detected by radiological examination, with no improvement after 2–4 weeks of treatment for common bacterial pneumonia (1,367 out of 4,015 cases, accounting for 34.05%); (2) patients with a history of TB treatment (either cured or not fully cured) and sputum-negative results (371 out of 4,015 cases, accounting for 9.24%); (3) those with sputum-positive or molecular biology-positive results (2,261 out of 4,015 cases, accounting for 56.31%); and (4) patients with sputum-negative results (1,754 out of 4,015 cases, accounting for 43.69%).

#### 2.2.1 New patients

Individuals who have not received anti-TB medications, those who have incompletely undergone the standardized treatment regimen, and those who have experienced irregular therapy for less than a month [8].

#### 2.2.2 Retreated patients

Patients who have received irregular and inappropriate anti-TB treatment for a minimum of one month, as well as those with treatment failure and relapse [8].

#### 2.2.3 Symptomatic contacts

Patients with a history of close contact with infectious TB exhibit corresponding clinical manifestations (e.g., cough, phlegm, low-grade fever, and night sweats).

#### 2.2.4 Etiology positive

Etiological confirmation is positive, characterized by sputum-based positivity and/or molecular biology-based positivity.

#### 2.2.5 Sputum positive

The results of the sputum or bronchoalveolar lavage smear revealed a positive detection for acid-fast bacilli and/or *M. tuberculosis* cultures.

① Sputum or bronchoalveolar lavage fluid smears positive.

② *M. tuberculosis* culture positive.

#### 2.2.6 Molecular biology positive

① Xpert MTB/RIF positive.

② Targeted high-throughput sequencing positive.

#### 2.2.7 Etiology negative

No evidence of *M. tuberculosis* infection was found through sputum or bronchoalveolar lavage fluid smears, *M. tuberculosis* culture, Gene Xpert testing, metagenomic sequencing, or other methods.

#### 2.2.8 Sputum negative

A sputum or bronchoalveolar lavage smear was not found to be positive for acid-fast bacilli and *M. tuberculosis* cultures.

#### 2.2.9 Regional distribution

Regional distribution is two urban districts in Huzhou City (Wuxing District and Nanxun District, the designated hospital for TB diagnosis and treatment was Huzhou Central Hospital), three counties (Changxing County, Deqing County, Anji County, the designated diagnosis and treatment hospitals for TB were Changxing County People’s Hospital, Deqing County People’s Hospital, and Anji County second People’s Hospital, respectively), and surrounding rural areas.

### 2.3 TB drug resistance definition and detection methods

**Mono-drug resistance:** Resistant to only one first-line anti-TB drug.

**MDR-TB:** Resistant to at least isoniazid and rifampicin [1].

**XDR-TB:** In addition to MDR-TB, resistant to at least one fluoroquinolone and one of three second-line injectable drugs (capreomycin, kanamycin, and amikacin) [3].

### 2.4 Statistical analysis

All statistical analyses were performed using Free Statistics software version 2.0. Continuous variables were tested for normality, and normally distributed variables were expressed as mean and standard deviation. Categorical variables were described using frequencies and proportions. The drug resistance patterns of the TB population in the local area were analyzed, and stratified analysis of MDR-TB cases by age was performed. Additionally, multivariate analysis methods were used to explore the influencing factors of MDR-TB. A significance level of *P* < 0.05 was considered statistically significant. Free Statistics software and Adobe illustrator software were used in the drawing.

## 3 Results

### 3.1 Demographic and clinical characteristics of the study population

Among the TB population, 3.66% (147/4,015) had MDR-TB. Most patients with TB had a body mass index (BMI) greater than 18.5 (85.7%), were smokers (81.0%), male (79.6%), sputum smear positive (70.7%), and farmers (66.0%), and resided in urban areas (62.6%). Regarding treatment, 87.1% of the patients were initially treated, and 12.9% were retreated. Among the MDR-TB population, 79.6% were male, 20.4% were female, 50.3% were ≤50 years of age, and 49.7% were >50 years of age. Most patients with MDR-TB were new cases (87.1%), had a smoking history (81.0%) and sputum smear positive (70.7%), and were farmers (66.0%). Among patients with TB having comorbidities, 26.5% (39/147) had diabetes, 15.0% (22/147) had a history of pneumoconiosis, and 2.0% (3/147) had human immunodeficiency virus (HIV). The additional baseline data are presented in Table 1.

**Table 1:** Demographic and clinical characteristics of TB and MDR-TB

Variables	Total (*n* = 4,015)	TB (*n* = 3,868)	MDR-TB (*n* = 147)	*P*
**Gender,** ***n*** **(%)**				0.008
Male	2,798 (69.7)	2,681 (69.3)	117 (79.6)	
Female	1,217 (30.3)	1,187 (30.7)	30 (20.4)	
**Residence,** ***n*** **(%)**				0.930
Urban	2,499 (62.2)	2,407 (62.2)	92 (62.6)	
Rural	1,516 (37.8)	1,461 (37.8)	55 (37.4)	
**Sputum smear,** ***n*** **(%)**				<0.001
Negative	1,754 (43.7)	1,711 (44.2)	43 (29.3)	
Positive	2,261 (56.3)	2,157 (55.8)	104 (70.7)	
**Type of TB treatment,** ***n*** **(%)**				0.089
New cases	3,654 (91.0)	3,526 (91.2)	128 (87.1)	
Retreated cases	361 (9.0)	342 (8.8)	19 (12.9)	
**Poverty,** ***n*** **(%)**				<0.001
No	3,127 (77.9)	2,996 (77.5)	131 (89.1)	
Yes	888 (22.1)	872 (22.5)	16 (10.9)	
**Hygienic condition,** ***n*** **(%)**				0.168
Well	2,827 (70.4)	2,716 (70.2)	111 (75.5)	
Poor	1,188 (29.6)	1,152 (29.8)	36 (24.5)	
**Smoking,** ***n*** **(%)**				**0.013**
No	1,126 (28.0)	1,098 (28.4)	28 (19.0)	
Yes	2,889 (72.0)	2,770 (71.6)	119 (81.0)	
**Age,** ***n*** **(%)**				0.942
≤50	2,033 (50.6)	1,959 (50.6)	74 (50.3)	
>50	1,982 (49.4)	1,909 (49.4)	73 (49.7)	
**BMI,** ***n*** **(%)**				0.015
≤18.5	351 (8.7)	330 (8.5)	21 (14.3)	
>18.5	3,664 (91.3)	3,538 (91.5)	126 (85.7)	
**Occupations,** ***n*** **(%)**				<0.001
Farmer	2,191 (54.6)	2,094 (54.1)	97 (66)	
Worker	561 (14.0)	546 (14.1)	15 (10.2)	
Unemployment	427 (10.6)	411 (10.6)	16 (10.9)	
Retirement	623 (15.5)	620 (16.0)	3 (2.0)	
Others	213 (5.3)	197 (5.1)	16 (10.9)	
**Comorbidities,** ***n*** **(%)**				<0.001
No	3,535 (88.0)	3,460 (89.4)	75 (51.0)	
Diabetes	285 (7.1)	246 (6.4)	39 (26.5)	
Pneumoconiosis	131 (3.3)	109 (2.8)	22 (15.0)	
HIV	37 (0.9)	34 (0.8)	3 (2.0)	
Others	27 (0.7)	19 (0.5)	8 (5.4)	

**Figure 1:**
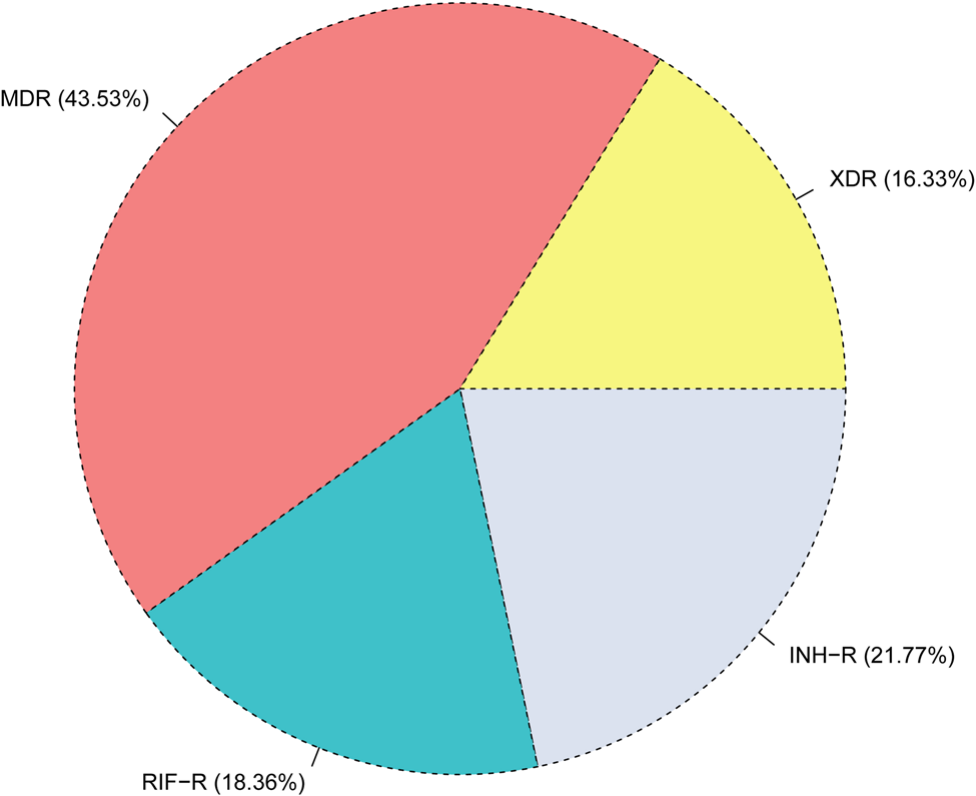
Drug resistance profile of 147 patients with DR-TB. Abbreviations: INH-R, isoniazid resistance; RIF-R, rifampin resistance; MDR-TB, multidrug-resistant tuberculosis; XDR-TB, extensively drug-resistant tuberculosis.

The drug resistance pattern is depicted in Figure 1, with 21.77% (32 cases) resistant to isoniazid, 18.37% (27 cases) resistant to rifampicin, 43.54% (64 cases) presenting with MDR, and 16.33% (24 cases) exhibiting XDR-TB (Figure 1).

### 3.2 Analysis of correlated variables in the occurrence of MDR-TB

**Figure 2:**
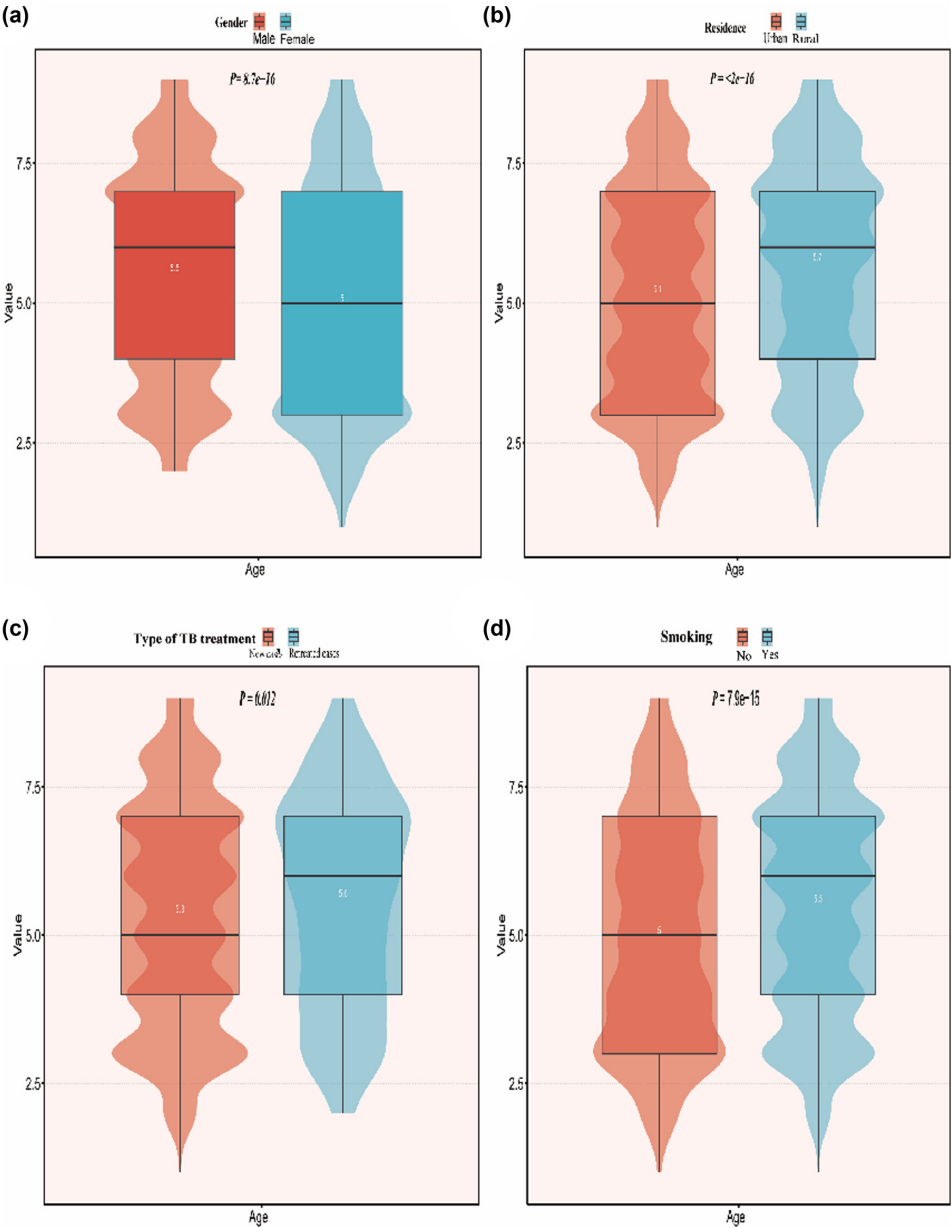
Comparison of different age groups with respect to gender, place of residence, type of TB treatment, and smoking status. The age of TB patients was divided into less than or equal to 10 years old, 10–20 years old, 20–30 years old, 30–40 years old, 40–50 years old, 50–60 years old, 60–70 years old, 70–80 years old, and more than or equal to 80 years old, and then, statistical analysis was performed according to gender (a), residence (b), type of TB treatment (c), and smoking or not (d).

The detection rate of DR-TB was higher in males than in females in all age groups. Among women, MDR-TB detection is highest in the age group 30–40 years, compared to men, where MDR-TB detection peaks between the ages of 50–70 years. The prevalence of MDR-TB is higher in the 20–70 age group in both rural and urban areas. The age group with an increased incidence of MDR-TB associated with retreatment is 50–70 years. Similarly, the age groups with increased rates of resistance associated with initial treatment were 20–30 and 50–60 years, respectively. The smoking group had the highest incidence of MDR-TB in the 60–70 year age group compared to the non-smoking group (Figure 2a–d).

### 3.3 Multivariate analysis of TB individuals with MDR-TB

Multivariate logistic regression analysis was performed to identify the risk factors for MDR-TB. The analysis was conducted while adjusting for confounding variables. The results showed that the risk of developing MDR-TB was 1.69 times higher in patients who had a smoking history than in those without (odds ratio [OR] = 1.69, 95% confidence interval [CI]: 1.11–2.56, *P* = 0.014). Besides, the risk of developing MDR is 0.58 times higher in women than in men (OR = 0.58, 95% CI: 0.39–0.87, *P* = 0.009). Moreover, the risk of progressing to MDR-TB with a positive sputum smear was 1.93 times higher than with a negative one (OR = 1.93, 95% CI: 1.35–2.77, *P* < 0.001). Additionally, the risk of MDR-TB in individuals as others was 0.61 times higher than in those occupations as farmers (OR = 0.61, 95% CI: 0.43–0.87, *P* = 0.006). Furthermore, the risk of developing MDR-TB in individuals with comorbidities was 9.17 times higher than in individuals without comorbidities (OR = 9.17, 95% CI: 6.5–12.93, *P* < 0.001). Finally, the risk of developing MDR-TB in patients with a BMI >18.5 was 0.55 times that of patients with a BMI of ≤18.5 (OR = 0.55, 95% CI: 0.34–0.88, *P* = 0.013). The additional multivariate logistic regression analysis data are presented in Table 2.

**Table 2:** Multivariate logistic regression analysis of TB individuals with MDR-TB

Variable	Total	*N* (%)	OR (95% CI)	*P*
**Gender,** ***n*** **(%)**				
Male	2,798	117 (4.2)	1(Ref)	
Female	1,217	30 (2.5)	0.58 (0.39–0.87)	0.009
**Residence,** ***n*** **(%)**				
Urban	2,499	92 (3.7)	1(Ref)	
Rural	1,516	55 (3.6)	0.99 (0.7–1.39)	0.939
**Sputum smear,** ***n*** **(%)**				
Negative	1,754	43 (2.5)	1(Ref)	
Positive	2,261	104 (4.6)	1.93 (1.35–2.77)	<0.001
**Type of TB treatment,** ***n*** **(%)**				
New cases	3,654	128 (3.5)	1(Ref)	
Retreated cases	361	19 (5.3)	1.53 (0.94–2.51)	0.090
**Poverty,** ***n*** **(%)**				
No	3,127	131 (4.2)	1(Ref)	
Yes	888	16 (1.8)	0.43 (0.25–0.73)	0.002
**Smoking,** ***n*** **(%)**				
No	1,126	28 (2.5)	1(Ref)	
Yes	2,889	119 (4.1)	1.69 (1.11–2.56)	0.014
**Age,** ***n*** **(%)**				
≤50	2,033	74 (3.6)	1(Ref)	
>50	1,982	73 (3.7)	1.02 (0.73–1.42)	0.912
**BMI,** ***n*** **(%)**				
≤18.5	351	21 (6)	1(Ref)	
>18.5	3,664	126 (3.4)	0.55 (0.34–0.88)	0.013
**Occupations,** ***n*** **(%)**				
Farmer	2,191	97 (4.4)	1(Ref)	
Others	1,804	50 (2.8)	0.61 (0.43–0.87)	0.006
**Comorbidities,** ***n*** **(%)**				
No	3,535	75 (2.1)	1(Ref)	
Yes	480	72 (16.9)	9.17 (6.5–12.93)	<0.001
**Lung diseases,** ***n*** **(%)**				
No	3,305	115 (3.5)	1(Ref)	
Yes	710	32 (4.5)	1.31 (0.88–1.95)	0.187

**Figure 3:**
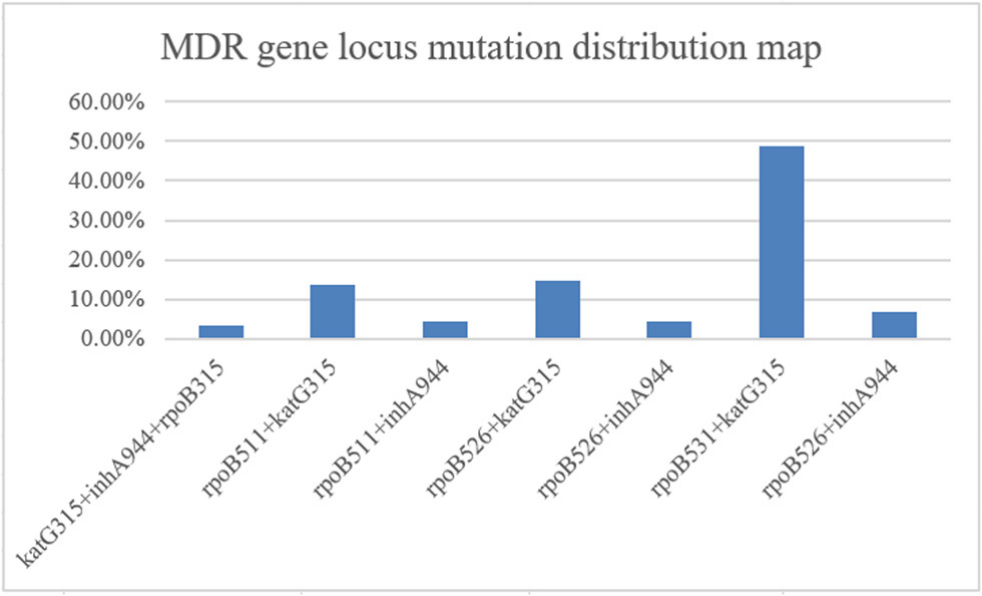
Mutation distribution map of the MDR gene locus. The mutations at the isoniazid-resistant loci katG and inhA were detected in 78.33% (94/120) and 19.17% (23/120) of the cases, respectively. The combination mutation katG + inhA was observed in 2.50% (3/120) of the cases. Among the mutations associated with rifampicin resistance, rpoB531 (58.26%, 67/115), rpoB526 (23.48%, 27/115), and rpoB511 (13.91%, 16/115) were the most prevalent. The mutation rates of rpoB + katG and rpoB + inhA in MDR-TB strains were 73.44% (47/64) and 21.88% (14/64), respectively.

The mutations at the isoniazid-resistant loci katG and inhA were detected in 78.33% (94/120) and 19.17% (23/120) of the cases, respectively. The combination mutation katG + inhA was observed in 2.50% (3/120) of the cases. Among the mutations associated with rifampicin resistance, rpoB531 (58.26%, 67/115), rpoB526 (23.48%, 27/115), and rpoB511 (13.91%, 16/115) were the most prevalent. The mutation rates of rpoB + katG and rpoB + inhA in MDR-TB strains were 73.44% (47/64) and 21.88% (14/64), respectively (Figure 3).

**Figure 4:**
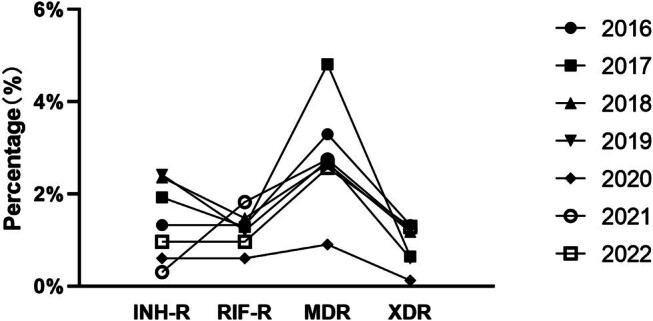
Trends of different drug-resistance patterns among DR-TB in Huzhou City, 2016–2022. Abbreviations: INH-R, isoniazid resistance; RIF-R, rifampin resistance; MDR-TB, multidrug-resistant tuberculosis; XDR-TB, extensively drug-resistant tuberculosis.

Notably, katG mutations were the most prevalent, with mutation rates exceeding 81.00% in 2017 and 2022. Between 2018 and 2019, the mutation rate of inhA significantly increased to 10.83% (13/120, *P* = 0.594). Furthermore, the combination mutation katG + inhA was detected in 2018 and 2019. The mutation frequency of rpoB531 isolates was 15.65% (18/115) in 2018 and 13.04% (15/115) in 2022, exhibiting a significant increase compared to 2020 (*P* < 0.05). Only three isolates presented the rpoB + katG + inhA mutation in 2018 and 2019. The mutation frequency of rpoB531 + katG315 in 2019 and 2021 was 35.94% (23/64, *P* = 0.007) and 23.44% (15/64, *P* = 0.048), respectively, exhibiting a significant increase compared to 2020 (4.68%, 3/64) (*P* < 0.05) (Figure 4).

## 4 Discussion

MDR-TB often results in protracted treatment, increased costs, and unfavorable reactions, posing challenges for managing relapses and difficult-to-treat cases. Consequently, comprehending the current epidemiology of drug resistance in this region is vital for achieving effective TB management and treatment success.

This study found that the prevalence of MDR-TB in the region was 3.66%. The study discovered that the detection rate of DR-TB in females is 0.58 times higher than in males. This suggests that males carry a heavier burden of DR-TB, potentially attributed to their lifestyle and smoking habits [9]. The study also found that smoking history was a potential risk factor for developing MDR, which is consistent with the view of several previous studies [10,11]. Furthermore, in this study, we found that the risk of MDR in sputum smear positive was 1.93 times higher than in sputum smear negative, which could contribute to the dissemination of TB and drug resistance, escalating the probability of resistance [3].

Notably, the higher drug resistance rate among young individuals [12] contests the misconception that they represent a low-risk group for DR-TB. This study determined that the risk of MDR-TB in individuals age greater than 50 years is approximately 1.02 times that of those less than or equal to 50 years old, in line with the findings of Wang et al. [13]; however, the difference was not statistically significant. This may be associated with factors such as population migration [14], crowded living environments [15], and unhealthy lifestyle habits, such as irregular sleep patterns and dietary habits [9], among those greater than 50. This highlights the need to screen new young TB patients for drug resistance. Some studies have revealed that the overall drug resistance rate in males is higher than in females [16]. However, our research indicates that females aged 20–30 years are also a population of concern, exhibiting a higher prevalence of MDR-TB. This may be associated with factors such as crowded living environments [12] and unhealthy lifestyles, including frequent late nights and lack of exercise [9]. Additionally, a history of previous treatment is a risk factor for MDR-TB, as some patients may develop resistance due to not following the prescription and irregular treatment [3]. This study found that the likelihood of MDR-TB in patients with comorbid diabetes and pneumoconiosis was approximately 9.17 times that of those without, suggesting that diabetes and pneumoconiosis may also be risk factors for developing MDR-TB, consistent with the findings of other researchers [16,17]. These results underscore the need for enhanced MDR-TB screening among pneumoconiosis and diabetes patients, particularly those with poor long-term blood sugar control. In this study, we found that the DR-TB notification rate of other occupations was 0.61 times higher than the rate of farmers. This suggested that the occupation of farmers and their working environment, which may be more exposed to dust, was associated with DR-TB [17], and several studies in other regions of China have also shown that farmers were a risk factor [18]. Some of the DR-TB patients come from the remote western region of China, where irregular management and irrational treatment may lead to the development of drug resistance. Furthermore, BMI ≤18.5 was a potential risk factor for the development of MDR-TB, consistent with the results of other studies [19]. Overall, these findings highlight the challenges in managing and controlling TB in China and emphasize the importance of preventing and intervening in the aforementioned risk factors.

Urban areas, due to their relatively dense population and high mobility, are more severely impacted by TB infections [20]. This study discovered that the likelihood of MDR-TB cases in rural areas is approximately 0.99 times that of urban areas, suggesting a higher incidence of MDR-TB in urban areas compared to rural areas. Moreover, the geographic mobility of individuals plays a crucial role in interpersonal transmission. Nelson et al. [14], utilizing genome sequence data and global positioning system coordinates, demonstrated that even within a province in South Africa, many cases of XDR-TB with minimal genetic variation (≤5 single-nucleotide polymorphisms) occurred among populations with a median distance of 108 km, emphasizing the role of migration between urban and rural areas in the transmission of TB. In the case of MDR-TB in this region, some cases originated from migrant workers from southwestern regions such as Yunnan and Guizhou, while others originated from Tibet. Interprovincial movement of individuals may have contributed to interpersonal transmission.

Although significant associations between resistance to isoniazid and rifampicin and mutations in the *M. tuberculosis*-resistant genes katG, inhA, rpoB, and ahpC have been observed, the location and frequency of these mutations vary across different regions [21]. For instance, the mutation rate of rpoB531 reaches as high as 64.8% in the Kyrgyz Republic [22], while the mutation rate of katG315 escalates up to 71.9% in Hainan Province, China [23], such regional differences in the frequency of mutations associated with drug resistance might mirror the molecular diversity of DR-TB strains prevalent in geographically distinct areas. Closely monitoring mutation patterns in these hotspot regions is vital for the timely detection of clinical TB drug resistance. The predominant gene mutations linked to MDR-TB transmission in this region are primarily located in codon KatG 315 and codon rpoB531, which is consistent with previous reports [24]. A comprehensive understanding of the mutation mechanisms in these two codons associated with resistance and their inhibition is essential for curbing the spread of MDR-TB.

## 5 Limitations

This study has several limitations. First, being a retrospective study, it faces some limitations in terms of data availability. Second, in the earlier years, most of our drug resistance locus testing was phenotypic drug resistance testing, classifying phenotypic resistance as “resistant” or “susceptible.” Future research designs will need to provide detailed descriptions of amino acid missense mutations and their corresponding single nucleotide polymorphism mutations for all drug-resistant isolates, facilitating the analysis of different clinical types of drug-resistant isolates. Third, information about the educational background, socioeconomic status, and living conditions of the patients involved in this study is incomplete. These factors can to some extent influence the prevalence of DR-TB. Fourth, the region is adjacent to neighboring developed cities, and some patients prefer to go to the nearest big cities such as Hangzhou and Shanghai, which may affect the accuracy of the epidemiological survey data in the region. Therefore, well-designed future studies should comprehensively elucidate the characteristics and risk factors of MDR-TB. Moreover, the limited number of MDR-TB cases in our region may hinder the representativeness of the sample. Therefore, future research should focus on conducting follow-up studies on a larger population of MDR-TB individuals through prospective investigations.

## 6 Conclusion

The prevalence of MDR-TB in the TB -affected population of the region was found to be 3.66%, underscoring the pressing need to enhance surveillance and management efforts, particularly among male patients, smoking history, sputum smear positive, farmer, BMI ≤18.5, retreated cases, those combination of diabetes or pneumoconiosis. Moreover, a focused approach on identifying mutations in the rpoB531 and KatG 315 genes could significantly contribute to early intervention in MDR-TB cases. These findings offer valuable epidemiological insights for effectively preventing and controlling TB within this specific region.
